# The sodium-glucose cotransporter 2 inhibitor luseogliflozin can suppress muscle atrophy in Db/Db mice by suppressing the expression of *foxo1*

**DOI:** 10.3164/jcbn.18-114

**Published:** 2019-05-18

**Authors:** Takuro Okamura, Yoshitaka Hashimoto, Takafumi Osaka, Takuya Fukuda, Masahide Hamaguchi, Michiaki Fukui

**Affiliations:** 1 Department of Endocrinology and Metabolism, Graduate School of Medical Science, Kyoto Prefectural University of Medicine, 465 Kajii-cho, Kamigyo-ku, Kyoto 602-8566, Japan; 2 Department of Diabetology, Ayabe City Hospital, Ayabe 623-0011, Japan

**Keywords:** sodium glucose cotransporter-2 inhibitor, luseogliflozin, muscle atrophy, *foxo1*, sarcopenia

## Abstract

We investigated the effect of the sodium glucose cotransporter-2 inhibitor (SGLT-2i) luseogliflozin on skeletal muscle. Eight-week-old mice were fed a standard diet or the standard diet with added luseogliflozin for 8 weeks. The mice were divided into the following four genotype/dietary groups: Db/m mice without SGLT-2i, Db/m mice with SGLT-2i inhibitor, Db/Db without SGLT-2i, and Db/Db with SGLT-2i. Among the mice with and without SGLT-2i, the ratio of soleus and plantaris muscle to body weight in the Db/Db mice was significantly lower than that in the Db/m mice. The cross-sectional area of soleus muscle in the Db/Db mice without SGLT-2i was significantly higher than that in the Db/Db mice with SGLT-2i. The expression of *foxo1* in soleus muscle of the Db/Db mice was significantly higher than that of the Db/m mice, and the *foxo1* expression of the Db/Db mice with SGLT-2i was significantly lower than that of the mice without SGLT-2i. The fluorescence intensity of *foxo1* in the Db/Db mice fed SGLT-2i was significantly lower than that in the Db/Db mice without SGLT-2i. The administration of luseogliflozin resulted in the suppression of both the increased *foxo1* expression and the reduced muscle cross-sectional area in the soleus muscle of Db/Db mice.

## Introduction

The numbers of individuals with type 2 diabetes are rapidly increasing worldwide. Complications of type 2 diabetes reduce a person’s quality of life, and they add a heavy burden to the medical economy.^(1)^ The prevention of the progression of diabetic complications is thus an important task. In recent years, muscle atrophy has been thought of as a complication of diabetes.^(2)^ It has become clear that muscle atrophy, i.e., sarcopenia, and sarcopenic obesity are strongly associated with dietary pattern or metabolic disorder.^(3,4)^ In fact, we demonstrated that muscle atrophy is present in diabetic patients.^(5,6)^ Muscle atrophy is also a risk factor for both decreased daily life activity and mortality.^(7,8)^

Several sodium glucose cotransporter-2 inhibitors (SGLT2i) have recently become available as anti-diabetic medications, and some of them have been reported to reduce the risk of incident cardiovascular disease.^(9,10)^ The effects of SGLT2i on body composition have been described,^(11,12)^ but the mechanisms underlying these effects on muscle have been unclear. We conducted the present study to investigate the effects of the SGLT2i luseogliflozin on muscle in Db/Db mice. We evaluated muscle atrophy using cross-sectional areas of muscle because this method has been often used as the best objective indicator of muscle atrophy.^(13,14)^ We also evaluated the changes in gene expression in skeletal muscle following the administration of SGLT2i. The genes *mstn*, *pgc1a*, and *foxo1* are related to muscle atrophy.^(15–17)^ We focused on *foxo1* in this study because the *foxo1* expression of skeletal muscle in individuals with diabetes is accelerated, and this suppresses the glucose utilization and lipid synthesis in skeletal muscle.^(18,19)^

## Materials and Methods

### Animals and experimental design

**Fig. 1:**
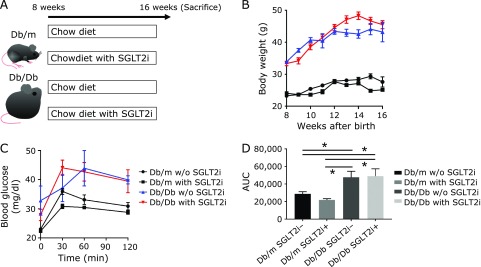
The SGLT2i luseogliflozin did not change the body weight of the mice or improve their impaired glucose tolerance. (A) Outline of the feeding and sacrifice protocol. (B) Body weight changes. (C, D) iPGTT results and the area under the curve of iPGTT. Data are mean ± SEM. *p<0.01 by *t* test.

All experimental procedures were approved by the Committee for Animal Research, Kyoto Prefectural University of Medicine. Six-week-old male non-diabetic heterozygous Db/m mice and 6-week-old male diabetic homozygous Db/Db mice were purchased from Shimizu Laboratory Supplies (Kyoto, Japan). Starting when the mice were 8 weeks old, they were fed either a standard diet (SD; 344.9 kcal/100 g, fat kcal 4.6%; CLEA Japan, Tokyo, Japan) or the same standard diet with the SGLT2i luseogliflozin added (0.01% w/w in chow) for 8 weeks. We divided the mice into the following four groups: (1) Db/m without (w/o) SGLT2i, (2) Db/m with SGLT2i, (3) Db/Db w/o SGLT2i, and (4) Db/Db with SGLT2i. At 16 weeks old, after an overnight fast, all of the mice were killed by the administration of a combination anesthetic: 0.3 mg/kg of medetomidine, 4.0 mg/kg of midazolam, and 5.0 mg/kg of butorphanol (Fig. 1A).^(20)^

### Glucose tolerance tests

Intraperitoneal glucose tolerance tests (iPGTTs) (2 g/kg) were performed in other 16-week-old mice that had been fasted for 5 h. Plasma glucose was measured from the tail vein using a glucometer (Gultest Neo Alpha; Sanwa Kagaku Kenkyusho, Nagoya, Japan).

### Tissue collection and histological assessment of murine soleus and plantaris muscles

We used the soleus and plantaris muscles for the muscle samples.^(21)^ The soleus muscle was either fixed with 10% buffered formaldehyde for the histological examination or immediately frozen in QIAzol Lysis reagent (Qiagen, Venlo, Netherlands) for mRNA extraction. We measured the weight and cross-sectional area of soleus and plantaris muscles of the four groups of mice described above. In this study, we used the anatomical cross-sectional area, which is the cross-sectional area of a muscle perpendicular to its longitudinal axis of soleus muscle.^(22)^

Soleus muscle sections were prepared and stained with hematoxylin and eosin or a monoclonal *foxo1* (C29H4) antibody (Cell Signaling Technology, Beverly, MA) as a primary antibody, and a Texas-red-conjugated anti-mouse secondary antibody (Jackson ImmunoResearch, West Grove, PA). Nuclei were stained with DAPI (Sigma-Aldrich, St. Louis, MO). Images were captured with a fluorescence microscope (BZ-X710, Keyence, Osaka, Japan), and the fluorescence intensity of the muscle tissue and the cell nuclei numbers were analyzed using Image J software. We measured the weights of the soleus and plantaris muscles and the cross-sectional areas of soleus muscle of the mice in the four groups described above. All images acquired using the BZ-X710 microscope and the cross-sectional areas of soleus muscle were measured using BZ-X analyzer software (Keyence).

### Gene expression in soleus muscle

The soleus muscle of fasting mice were resected and immediately frozen using liquid nitrogen and homogenized in ice-cold QIAzol Lysis reagent, and total RNA was isolated as described in the manufacturer’s instructions. We reverse-transcribed the total RNA (0.5 µg) by using a High-Capacity cDNA Reverse Transcription Kit (Applied Biosystems, Foster City, CA) for first-strand cDNA synthesis utilizing an oligonucleotide dT primer and random hexamer priming according to the manufacturer’s recommendations. The reverse transcription (RT) reaction was performed for 120 min at 37°C, and the inactivation of RT was performed for 5 min at 85°C.

The mRNA expression levels of *foxo1*, *myog*, *mstn*, *myod*, *pgc1a* and *ppara* were quantified using a real-time reverse transcription-polymerase chain reaction (RT-PCR). The relative expression levels of each targeted gene was normalized to the *gapdh* threshold cycle (CT) values and quantified using the comparative threshold cycle 2^−ΔΔ*C*_T_^ method as described.^(23)^ Signals from Db/m mice without SGLT2i feeding were assigned a relative value of 1.0. The RT-PCR was performed using TaqMan Fast Advanced Master Mix (Applied Biosystems) according to the manufacturer’s instructions. The following PCR conditions were used: 1 cycle for 2 min at 50°C and 20 s at 95°C, followed by 40 cycles for 1 s at 95°C, and s0 s at 60°C.

### Statistical analysis

We analyzed the data using the JMP ver. 13.0 software (SAS, Cary, NC), and *p* values <0.05 were considered signiﬁcant. Student’s *t* test was used to compare the differences between pairs of groups.

## Results

### Effect of SGLT-2i on body weight and glucose homeostasis

After the 8-week dietary treatment, the body weight and blood glucose in the two groups of Db/Db mice (those with and w/o the SGLT2i) were significantly higher than those of the two groups of Db/m mice. However, no significant reduction in body weight and no improvement in impaired glucose tolerance were observed following the administration of SGLT-2i (Fig. 1B–D).

### Effect of SGLT-2i on skeletal muscle

**Fig. 2:**
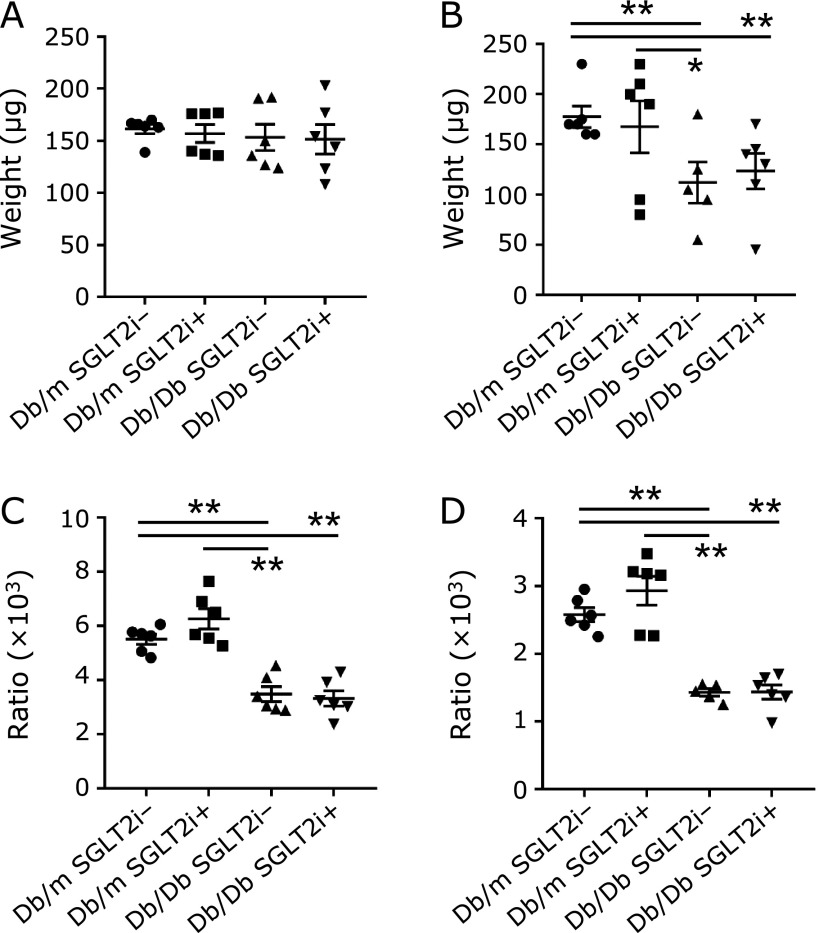
Luseogliflozin in the diet did not change the muscle weights of the mice. (A) Plantaris muscle weights (*n* = 6). (B) Soleus muscle weights (*n* = 6). (C) Ratio of plantaris muscle to body weight (*n* = 6). (D) Ratio of soleus muscle to body weight (*n* = 6). Data are mean ± SEM. *p<0.05, **p<0.01 by *t* test.

**Fig. 3:**
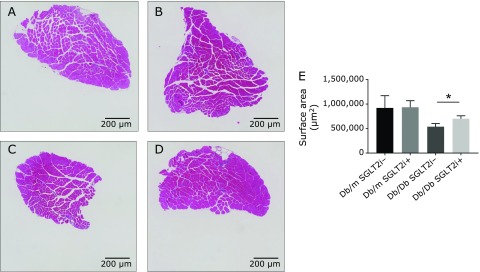
Histological assessment of the soleus muscle. Luseogliflozin increased the cross-sectional area of soleus muscle. (A–D) Cross-sections of soleus muscle. (A) Db/m without SGLT2i. (B) Db/m with SGLT2i. (C) Db/Db without SGLT2i. (D) Db/Db with SGLT2i. Scale bar, 200 µm. (E) Cross-sectional area of soleus muscle. Data are mean ± SEM. *p<0.01 by *t* test.

In the mice treated with and without SGLT-2i, the weight of the soleus muscle of the Db/Db mice was significantly lower than that in the Db/m mice, whereas the weight of the plantaris muscle did not show a significant difference between the Db/Db and Db/m mice (Fig. 2A and B). Additionally, among the mice treated with and without SGLT-2i, the plantaris and soleus muscle to body weight ratio in the Db/Db mice was significantly lower than that in the Db/m mice (Fig. 2C and D). The cross-sectional area of soleus muscle in the Db/Db mice without SGLT-2i was significantly less than that in the Db/Db mice with SGLT-2i (Fig. 3A–E).

### SGLT-2i suppressed foxo1 expression in muscle

**Fig. 4:**
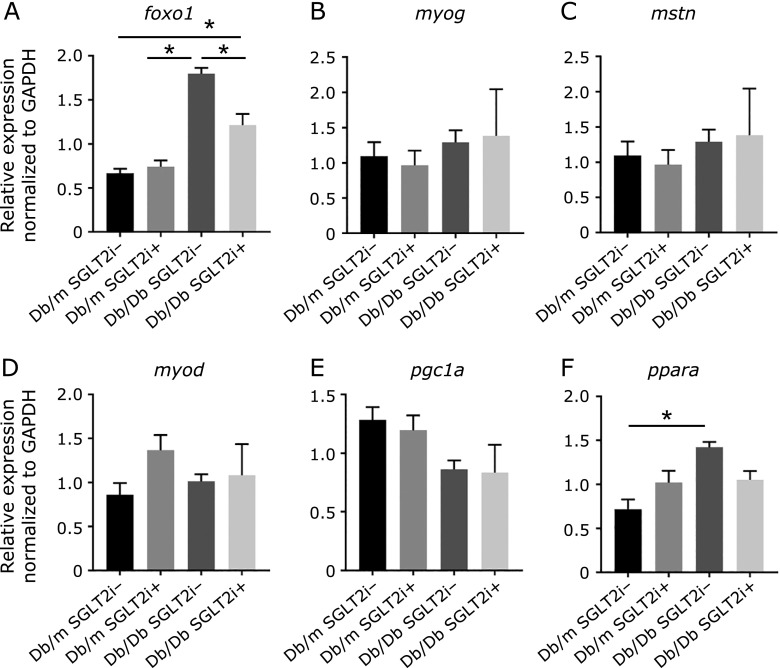
Luseogliflozin significantly suppressed the expression of *foxo1* in Db/Db mice. RT-PCR analysis of gene expression in soleus muscle. (A) *foxo1*. (B) *myogenin*. (C) *myostatin*. (D) *myod*. (E) *pgc1a*. (F) *ppara* (*n* = 6). Data are mean ± SEM. *p<0.05 by *t* test.

**Fig. 5:**
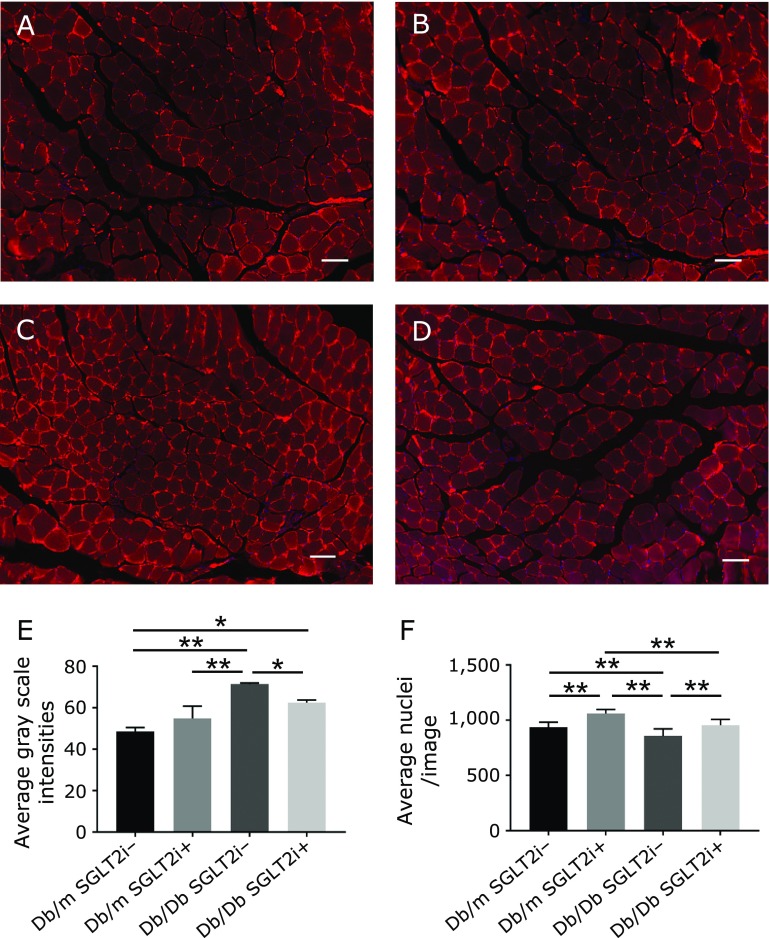
Luseogliflozin significantly suppressed the expression of *foxo1* in muscle tissue. Immunofluorescence of *foxo1* of soleus muscle. Immunostainings are shown. (A) Db/m without SGLT2i. (B) Db/m with SGLT2i. (C) Db/Db without SGLT2i. (D) Db/Db with SGLT2i. Scale bar, 50 µm. (E) Fluorescence intensity. (F) Number of cell nuclei per image. Data are mean ± SEM. *p<0.05, **p<0.01 by *t* test.

Our RT-PCR analyses revealed that the *foxo1* expression in skeletal muscle of the Db/Db mice was significantly higher than that of the Db/m mice (Fig. 4A). However, the *foxo1* expression in skeletal muscle of the Db/Db mice with SGLT-2i was significantly lower than that in the mice without SGLT-2i (Fig. 4A). The administration of SGLT-2i did not change the expressions of any other genes in the Db/m and Db/Db mice (Fig. 4B–F). In addition, the immunostaining of soleus muscle tissues demonstrated that the fluorescence intensity of *foxo1* in the Db/Db w/o SGLT2i group was significantly higher than that of the Db/Db with SGLT2i group (Fig. 5A–E). Moreover, the number of cell nuclei per image in both the Db/m mice and the Db/Db mice treated with SGLT-2i were higher than those of the mice w/o SGLT-2i (Fig. 5F).

## Discussion

Our findings demonstrated that the *foxo1* expression in skeletal muscle of Db/Db mice is higher than that of Db/m mice and that an SGLT2i, luseogliflozin, suppressed this higher *foxo1* expression in skeletal muscle of Db/Db mice. Increased *foxo1* expression in skeletal muscle was reported to be associated with muscle atrophy.^(24,25)^ *Foxo1* could affect several metabolic pathways. Among them, proteolysis regulated by the ubiquitin-proteasome pathway, autophagy, and the repression of protein synthesis are dominant processes of muscle atrophy.^(17,26)^

In addition, *foxo1* has been thought to have a pivotal role in glycolysis in muscle. In fact, increased *foxo1* expression resulted in the upregulation of *pdk4* expression, which suppresses the glycolytic pathway.^(18)^ Increased *foxo1* expression represses the expression of *srebp1c*, which is mediated by nuclear receptors (such as liver X receptor and retinoid X receptor), and it upregulates the biosynthesis of fatty acid in skeletal muscle.^(19)^ Therefore, increased *foxo1* expression in skeletal muscle suppresses glucose utilization and lipid synthesis.

In the present study, the ratio of plantaris and soleus muscle to body weight in the Db/Db mice were significantly lower than that in the Db/m mice. Moreover, the cross-sectional area of soleus muscle in the Db/Db mice treated with SGLT2i was significantly higher than that of the mice w/o SGLT2i.

This study has some limitations. First, the sample size was small. Second, we did not investigate the biological mechanism of luseogliflozin *in vitro*. This issue is very important and should be addressed in future studies.

## Conclusion

Taken together, our present findings suggest that increased *foxo1* expression in skeletal muscle is associated with the muscle atrophy of Db/Db mice. This is the first study to demonstrate the increased expression of *foxo1* in muscle tissue of Db/Db mice. The administration of luseogliflozin resulted in the suppression of both the increased *foxo1* expression and the reduced muscle cross-sectional area in the soleus muscle of Db/Db mice. Further studies investigating the association between the effect of an SGLT-2i on muscle and *foxo1* in muscle are needed.

## Abbreviations

iPGTT: intraperitoneal glucose tolerance tests
RT: reverse transcription
RT-PCR: real-time reverse transcription-polymerase chain reaction
SD: standard diet
SGLT-2i: sodium glucose cotransporter-2 inhibitor
w/o: without
